# Learning curve of digital intraoral scanning – an in vivo study

**DOI:** 10.1186/s12903-020-01278-1

**Published:** 2020-10-19

**Authors:** Ivett Róth, Alexandra Czigola, Gellért Levente Joós-Kovács, Magdolna Dalos, Péter Hermann, Judit Borbély

**Affiliations:** Department of Prosthodontics, Semmelweis University, Szentkiralyi Street 47, 1088 Budapest, Hungary

**Keywords:** Digital impression taking, Intraoral scanner, Learning curve

## Abstract

**Background:**

The spread of digital technology in dentistry poses new challenges and sets new goals for dentists. The aim of the present in vivo study was to determine the learning curve of intraoral scanning described by (1) scanning time and (2) image number (count of images created by intraoral scanner during the scanning process).

**Methods:**

Ten dental students of Semmelweis University took part in the study. Dental students took digital study impressions using a 3Shape Trios 3® (3Shape, Copenhagen, Denmark) intraoral scanning device. Each student took 10 digital impressions on volunteers. Volunteer inclusion criteria included full dentition (except for missing third molars) and no prosthetic/restorative treatment. Digital impression taking was preceded by tuition consisting of both theoretical education and practical training. Digital impressions were taken of the upper and lower arches, and the bite was recorded according to the manufacturer's instructions. Total scanning times and image numbers were recorded.

**Results:**

The difference in scanning time between the first and the tenth digital impressions was significant (*p* = 0.007). The average scanning time for the first impressions was 23 min 9 s; for the tenth impressions, it was 15 min 28 s. The difference between the scanning times of the first and the tenth procedures was 7 min 41 s. The average image count for the first impressions was 1964.5; for the tenth impressions, it was 1468.6. The image count difference between the first and the tenth procedures was 495.9. The image count versus sequential number of measurement curve shows an initial decreasing tendency followed by a trough around the sixth measurement and a final increasing phase.

**Conclusion:**

Our results indicate an association between the sequential number of measurements and the outcome variables. The drop in scanning time is probably explained by a practice effect of repeated use, i.e. the students learned to move the scanning tip faster. The image count first showed a decreasing tendency, and after the sixth measurement, it increased; there was no consistent decline in mean scan count. Shorter scanning times are associated with poorer coverage quality, with the operator needing to make corrections by adding extra images; this manifests as the time function of image counts taking an increase after the sixth measurement.

## Background

The widespread use of digital technology is transforming our everyday lives: computers and digital devices offer easier, faster, and more economical alternatives to conventional methods. In recent years, dentistry has made progress with the integration of CAD/CAM (computer-aided design/computer-aided manufacturing) technology as well as many novel tools and methods. After CAD/CAM technology was introduced, it did not take long for dental applications to emerge. It was Dr. Francois Duret who created the first CAD/CAM restoration in 1983; he then demonstrated his system at the France Dental Association's international congress in 1985. At the Midwinter Meeting in 1989, Dr. Duret confected a crown in four hours live on stage [1]. In the last few years, several intraoral scanning devices have been introduced in the field of dentistry [2–10]. When it comes to implementing a system of direct digital workflow, a dentist must have access to an intraoral scanner [4]. Digital impression taking has benefits such as reduced gag reflex potential, decreased working time, no potential deformation of impression material or expansion of gypsum, real-time visualization, and easy repeatability [9, 11, 12]. However, intraoral scanning also has some limitations: some studies state that conventional impressions are a better solution for challenging prosthodontics (e.g. accuracy of long-span restorations on multiple implants) [13–15], difficult bite registration (many systems do not support the registration of dynamic occlusion), scanning fees in closed systems (the user has to pay for performing the scanning data), and costs (intraoral scanning systems are still expensive) [2]. Furthermore, these new methods have a learning curve: dentists are required to put in practice hours before they can use these devices effectively [16]. Learning is defined as “an enduring change in behavior or in the capacity to behave in a given fashion resulting from practice or other forms of experience” [17]. A learning curve is the representation of the rate of learning something over time or repeated instances in a visual form [18]. Concerning the introduction of new technologies or techniques in general medicine, several studies have determined the learning curves of users [19–21]. Intraoral scanning has been investigated in many studies compared with conventional impression taking [22–27]. Previous studies in the field of digital dentistry focused on the accuracy and effectiveness of intraoral scanners [3, 5, 6, 10, 11, 13, 14, 24] and the observations of well-trained dentists and dental students [22, 25]; however, little data are available about the proficiency of the person who is scanning. Students preferred digital impression over the conventional impression technique. Older clinicians were less passionate about digital innovations in dentistry due to their personal history of having used a conventional method for impression taking with good results for a long time [22, 25]. There have not been any standardized and randomized clinical studies assessing the learning curve of digital impression taking. For a practicing dentist, it is crucial to know the learning curve of taking digital impressions and the applicability of the scanner when considering investment in a new system. The learning process is represented by the reduction of the time required for taking digital impressions and the decrease in the number of images of the virtual model. The operation of intraoral scanner systems is based on optical scanning techniques (visible or amplified light beam). In our study, a Trios 3® (3Shape, Copenhagen, Denmark) intraoral scanner was used, which employs a visible light beam for imaging and operates on the basis of real-time image capturing technology (ultrafast optical sectioning technique). The ultrafast optical sectioning technique utilizes up to 1000 3D images to create geometries from real data [28]. Based on ultrafast optical sectioning, Trios 3® builds digital models by taking pictures and stitching them together. The first picture is used as a reference, and the others are connected to it with some overlap [23, 29]. The larger the number of such overlaps, the more inaccurate the virtual model. We should, therefore, try to create as few images as possible to obtain the full digital impression without any missed areas.

The aim of the present in vivo study was to determine the learning curve of intraoral scanning described by [1] scanning time and [2] image number (count of images created by intraoral scanner during the scanning process). The null hypothesis was that there is no association between the sequential number of measurements and the outcome variables (total scanning time and number of images).

## Methods

### Participant education

The approval for this study was given by the University Ethics Committee of Semmelweis University (SE TUKEB number: 61/2016). Dental students 6 to 10 semesters into their graduate dental studies at Semmelweis University with no experience of digital impressions took part in this study as examiner students. The study was preceded by education covering both theory and practice.

During the theoretical part of the education, a presentation was held by a dentist experienced in scanning, and an educational video (made by the research team) was viewed. The presentation was about the types, structure, operating principles, and indication areas of digital scanners. The intraoral scanner was introduced in detail as it was to be used in the study. The video focused on the practical application of the scanner. In the video, the process of taking a digital impression was introduced step by step. This was followed by practical training where each student took a digital impression with the intraoral scanner of a lower and upper jaw model in an articulator with occlusion recording.

### Participants of the study

Participants of the study included examiner students (students who took digital impressions), volunteers, and supervisor dentists. Ten dental students were involved in intraoral scanning in pairs assisting each other. Students had no previous experience with intraoral scanning. During scanning, supervision was granted by a dentist (supervisor) with experience in digital impression taking. Each examiner student took 10 scans. The scans were performed separately from one another. The whole data collection procedure was performed between June 2016 and September 2017.

The patient’s various individual characteristics such as salivary flow rate or extent of mouth opening can affect the speed of the digital scanning procedure. Volunteer subject inclusion criteria included full dentition (except for missing third molars), good oral hygiene, at least 18 years of age, intact hard and soft tissue (no decay or tooth extraction sockets), and normocclusion (Angle I). The exclusion criteria were history of orthodontic treatment, dental implants, any prosthetic treatment (inlays/onlays or crowns), gingivitis or periodontitis.

The examiner students worked in pairs: one of them took the digital impression and the other one assisted (i.e. each examiner student took 10 digital impressions and assisted in another 10 procedures). The scanning student was on the right side, and the assisting student was on the left side of the volunteer. The scans were performed with patients in a supine position. All scans were performed with the help of a retractor (Optragate, Ivoclar Vivadent) to maximize accessibility. Dental lights were turned off while scanning.

### Intraoral scanner

As to the intraoral scanner (IOS), the same IOS device was used throughout the study. In line with a scanning protocol based on the manufacturer’s instructions, students took digital impressions with the USB version of the IOS, which can be connected to a high-performance laptop with a pen grip [2]. The software and hardware of the IOS are capable of capturing full-color models. This scanner is a powder-free device and operates on the confocal principle with the video recording method [2, 30]. Before starting to scan, the scanner was calibrated using the appropriate supplementary tips and calibration box. The software version 3Shape Trios Classic 1.3.4.6 was used.

### Digital impression taking

**Fig. 1:**
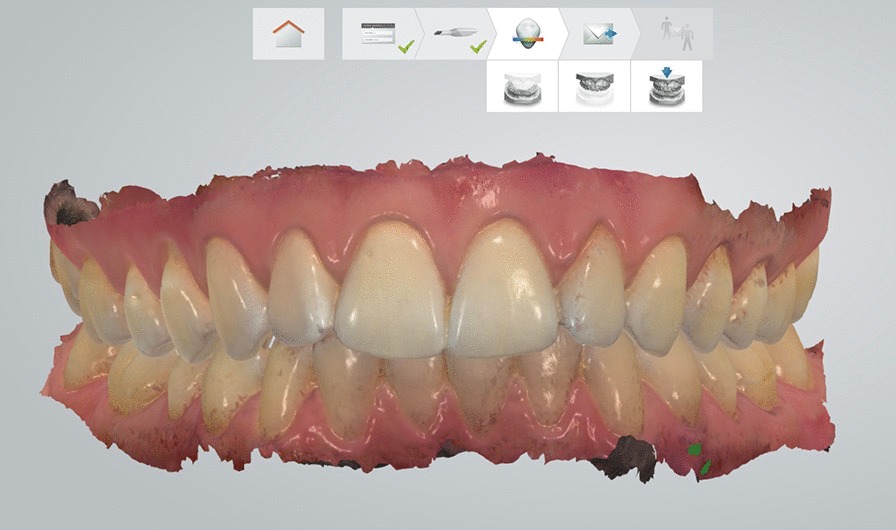
The virtual model was accepted if every surface of every tooth was scanned and the bite registration was successful

Diagnostic scans were taken after selecting the “Study model” icon on the control interface. First, patient data and the digital order form were completed. Scanning procedures were performed in accordance with the manufacturer's instructions and prior education. They were started on the upper jaw followed by the lower one. Scanning always began at the right second molar and continued along the arch all the way to the left second molar. The scanning sequence on the upper arch is occlusal then buccal and, finally, palatal surface, while on the lower jaw, the occlusal surface is followed by the lingual and buccal surfaces [31]. The next step was bite registration in intercuspidal position on both sides. During bite scan, the scanner tip was inserted at the buccal side of the teeth in the molar region and slowly moved in the mesial direction. After the upper and lower arches had been scanned, the virtual cast appeared on the screen. The virtual casts of the upper and lower arches were accepted if they included accurate scans of all surfaces of all teeth with 2–3 mm of gingival margin and no crack lines found. The quality of the scans was satisfactory if the software was able to line up the arches based on the bite registration scan [16] (Fig. 1). If a crack line appeared on the virtual cast, the procedure was repeated from the beginning (the virtual cast was deleted and a new intraoral scan was taken). In case of missing data (e.g. sporadic unscanned dental surface areas) the virtual cast was not deleted but additional images were taken. Irrelevant areas such as palatal soft tissue were removed.

### Registered data

Scanning time was measured with a stopwatch. Total scanning time was measured from the first step of recording patient data to the sending of the case to the lab. Total impression taking time included duration of data recording, scanning time required for complete scanning of the upper and lower arches, bite registration on both sides, and processing time of the scan. Image numbers of the upper and lower arches and bite registration were also recorded. Image number is the count of images created by intraoral scanner during scanning. The number of images appeared automatically in the upper left corner of the screen after scanning. The present study focused on the total required scanning time and the total count of images.

### Learning curve

**Fig. 2:**
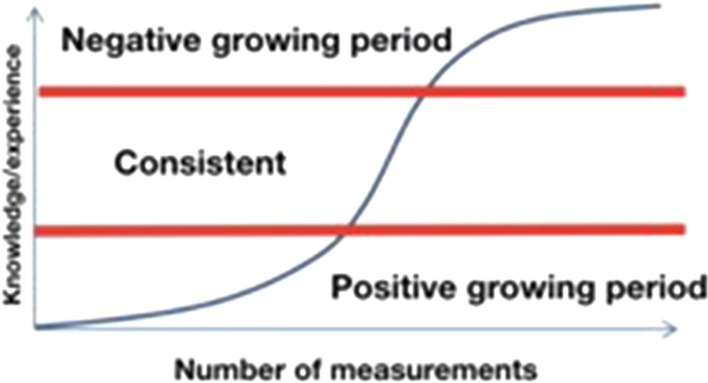
The classic-type learning curve

A learning curve is a visual representation of the rate of learning something over time or repeated experiences. Various types of learning curves exist; the classic type is an ascending sigmoid curve starting from learning level zero. The curve can be divided into three sections. During the positive growing period, the learning rate of the subjects is increasing constantly, followed by a middle section where the pace of learning is uniform. During the negative period, the learning rate of the subjects decreases and, finally, the curve ends in a flat phase (Fig. 2).

**Fig. 3:**
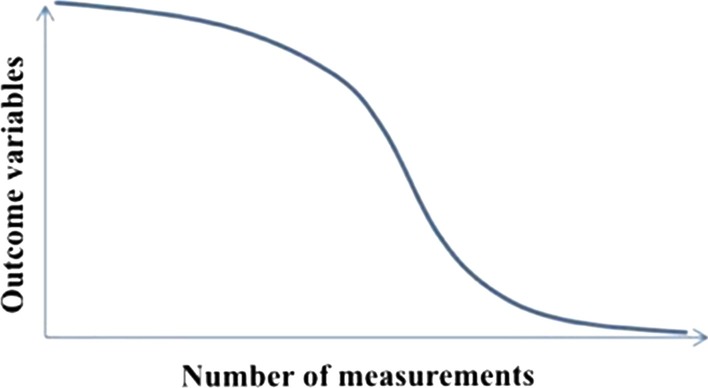
The inverse learning curve

If the repeated experiences (in this study, number of scan procedures completed) is represented on the *x* axis and the *y* axis represents resource costs, e.g. time or number of images required, the result is an inverse learning curve [17] (Fig. 3). In statistical evaluation, we can draw an inverse learning curve of the examiner students from the point of view of total scanning time and number of images.

### Statistical analyses

Statistical evaluation was carried out using the Stata package to fit random-effects generalized least-squares regression models of outcome variables (total scanning time and image count data) against the sequential number of measurements, a continuous explanatory variable analogous with learning stage. Relationship curvature was allowed by adding a squared term for measurement number if its effect was significant at α = 0.05. Hausman’s specification test was used to assess whether fitting a fixed-effects model was justified. Outcome variables were natural log-transformed to improve normality.

## Results

The null hypothesis stated that there was no association between the sequential number of measurements and the outcome variables (total scanning time and number of images). Based on our results, the null hypothesis was rejected since repeated use of intraoral scanner was associated with decreasing scanning times and image numbers.

**Fig. 4:**
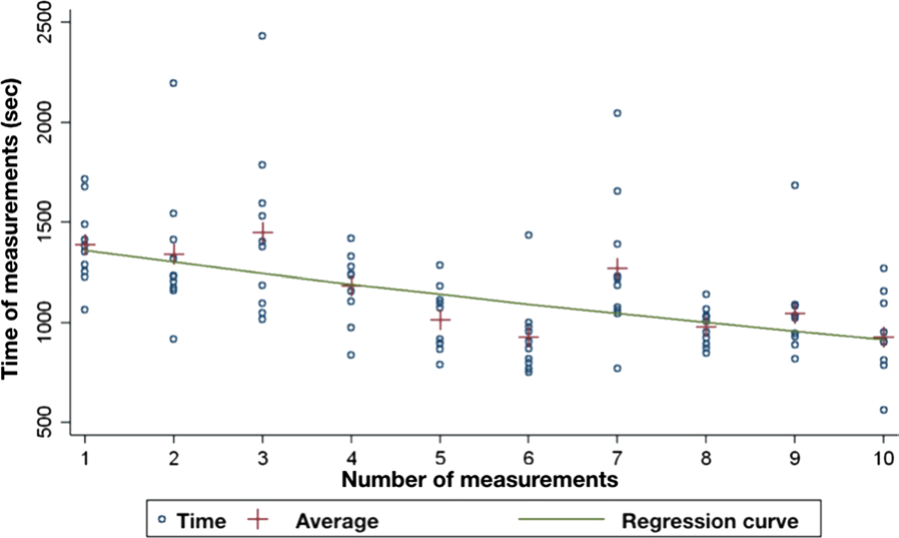
Regression curve of scanning time against measurement number

**Fig. 5:**
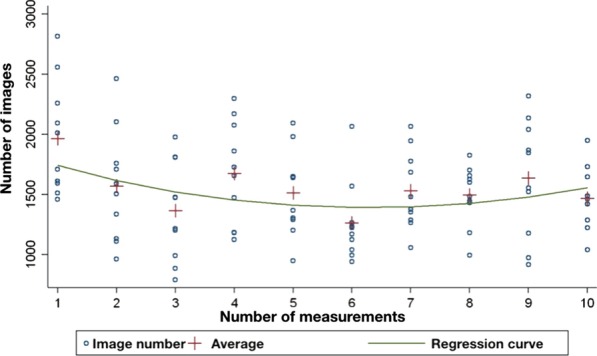
Regression curve of image count against measurement number

It can be seen from the 100 measurements that the average total impression taking time was 23 min 9 s for the first, and 15 min 28 s for the tenth scanning; the difference between the two (7 min 41 s) is significant (*p* = 0.007). The mean total image number for the first measurement was 1964.5; by the tenth measurement, it was 1468.6 (a difference of 495.9). There is no consistent decline in average image number. For the required scanning time, the learning curve fitted on the measured data is consistent with the second part of a classic learning curve (Fig. 4). As to image numbers, the curve has a trough around the sixth measurement and then rises again (Fig. 5). Within the limitations of our measurements, this curve is at the boundary of the middle and last thirds of the inverse learning curve.

## Discussion

Since the introduction of CAD/CAM technology, techniques for its initial two steps, direct and indirect impression taking, have been studied by several researchers [32–34]. Increased interest in intraoral scanners has induced an expanding volume of research. The accuracy and time efficiency of intraoral scanners, as well as the opinions of patients, dentists, students, and assistants, have been chosen by many researchers as their topic [24, 30, 35]. Numerous clinical studies on digital impression taking have been carried out by well-trained dentists, i.e. those who had used the examined scanners several times before [29, 34, 36–38]. Nevertheless, it is not mentioned how and for how long this practice was accumulated. Basically, the experience of scanner users determines the user acceptance of intraoral scanners [39, 40]. Many dentists refuse to use these new tools because of a long learning process. They believe that learning intraoral scanning will be just as difficult as the practice of traditional impression taking is for a student or a recently graduated dentist [41]. Investigating the learning process of how intraoral scanners are used is an important part of integrating them into everyday clinical practice. In the present study, our research group evaluated the effectiveness of intraoral scanning based on two parameters: scanning time and number of images. There was an association between the sequential number of measurements and the outcome variables as repeated intraoral scanner use resulted in decreasing scanning durations and a time-dependent progression of image numbers; the null hypothesis was therefore rejected. During digital impression taking, the average scanning time decreased due to practice. Our average scanning time observed at the tenth procedure (15 min 28 s, an improvement of 7 min 41 s over the first trial) is remarkably close to another research group’s mean scanning time findings (14 min 25 s) in a study where the flat phase of the curve was also not reached upon taking 10 digital impressions [42].

In our research, impressions were taken by dental students who had no previous experience with intraoral scanning. This allowed the assessment of learning speed in digital dentistry objectively. On the other hand, dental students tend to be open-minded when it comes to digital innovation. This is why their average scanning time turned out to be shorter than that of experienced dentists. For the required scanning time, the curve fitted on the measured data was located in the second part of a classic learning curve. Digital impression taking was preceded by tuition in two parts: theoretical education and practical training. In our research, the learning curve did not start from zero because of this training. The positive growing period was not shown in our curve. The inverse learning curve showed a decreasing tendency, but the flat phase was not reached in this study [43]. For comparison, the average time for conventional (two-step silicone) impression of the upper jaw is 7 min 30 s (excluding preparation time e.g. tray selection) [44]. Another research group evaluated the total treatment time for the conventional impression of maxillary and mandibular dental arches with polyether impression material (Impregnum, 3 M ESPE) using the monophase impression technique. The last step of the conventional technique was bite registration with polysiloxane bite registration material. The mean total procedure time of the conventional technique was about 10 min 5 s, which is less than our observed intraoral scanning time; this is probably explained by the conventional impressions having been taken by a well-trained clinician [45].

Image counts have not yet been fully explored in the literature in this research field. In our study, the image count first showed a decreasing tendency, and after the sixth measurement, it increased. Early in the learning process, examiner students moved the scanning tip slower, and the scanner made the appropriate number of images. The coverage quality was acceptable, and there was no need to take additional images. As a result, the image number showed a decreasing tendency through the first six measurements. Scanning speed was increased by practice from procedure to procedure, but the operator tended to make more mistakes. Unscanned areas appeared, which had to be corrected by adding new images; as a result, the scanning speed decreased but the image count increased after the sixth measurement. The average image number at the tenth procedure was 1468.6 for two arches and bite registration. A research group from Shanghai Jiao Tong University School of Medicine conducted a study in 2016. In their research, the average image number was 835 and the average scanning time was 4 min 58 s after 96 digital impressions of the entire upper jaw taken using a Trios 3® intraoral scanner. The procedures were confined to the upper jaw and were carried out by a well-trained dentist, which explains the difference between these results and ours [44]. There is no correlation between the number of images acquired and the accuracy of the digital impression. A high image count can lead to longer post-processing times but there is no evidence for decreasing the precision of the virtual cast. On the other hand, a low number of images can result in an insufficient digital impression*.* The number counter of the scanner is provided to reduce the risk of a longer processing time and hardware overheating, depending on the hardware specifications of the computer. To avoid hardware overheating, we used the Pod version of Trios 3® with the official laptop sold with the scanner.

There are some limitations of this study. We only determined the proficiency of the person who performed the scanning but did not investigate the precision of the later restoration. Furthermore, we used only one intraoral scanner. It operates using confocal laser technology and the data capture mode is video sequence [2]. There are many different types of scanners with different data capture principles which can result in varied outcomes. Another limitation was the low number of scans. We had a total of 100 digital impressions taken by 10 operators (10 scans by each examiner). If the number of scanning is increased, the flat phase can be reached. Moreover, the fact that examiner students worked in pairs (each examiner student took 10 digital impressions and assisted in another 10) could contribute to their learning curve as these students may have had an advantage as they have already seen the exam.

Furthermore, it would be interesting to compare the learning curves of students and experienced clinicians. Patient-related factors presented another limitation. Saliva flow, movement of the tongue or the patient, and limited oral space have a strong influence on scanning speed [46, 47]. In our study, the 10 volunteers who were scanned were selected based on our inclusion and exclusion criteria which were detailed in the paragraph “Participants of the study”. Furthermore, the volunteers were not independent from the study because they were also dental students. For this reason, they tolerated the scanning procedure better than real patients.

## Conclusions

The learning curve of intraoral scanning can be described in terms of scanning time and the image number of digital impressions. Based on our results, there was an association between the sequential number of measurements and these outcome variables. Scanning time decreased along with repeated use of the intraoral scanner. The last average scanning time was 15 min 28 s; the difference between the first and the last procedure was 7 min 41 s. Scanning time decreased because the students moved the scanning tip faster as a result of practice. Scanning speed increased but shorter scanning times were accompanied by poorer coverage quality; the operator had to make corrections by adding extra images. The image number first showed a decreasing tendency, and after the sixth measurement, it increased. There was no overall decline in mean scan count, the average of which at the tenth procedure was 1468.6.

Given the limits of this study, the flat phase of the learning curve was not reached because ten digital impressions were not enough to reach the average scanning time/image number of an experienced user; therefore, further measurements are necessary. This study observed a progressive increase in the scanning speed of digital impression taking within a short period of training in the digital impression method among dental students. Evaluating the operation of intraoral scanners is important for long term clinical application.

## Data Availability

The datasets used and/or analyzed in the current study are available from the corresponding author upon reasonable request.

## Abbreviations

CAD/CAM: Computer-aided design/computer-aided manufacturing
